# Zinc Oxide Nanoparticle-Induced Neurotoxicity: Underlying Molecular Mechanisms and Future Perspectives

**DOI:** 10.3390/toxics14010011

**Published:** 2025-12-20

**Authors:** Chun Chen, Xingyao Pei, Yonger Yu, Chang Gao, Jinran Wang, Rongyao Zhu, Shuxuan Liu, Shusheng Tang, Daowen Li

**Affiliations:** 1 State Key Laboratory of Veterinary Public Health and Safety, College of Veterinary Medicine, China Agricultural University, Beijing 100193, China; chenchun110212@163.com; 2 Tianjin Key Laboratory of Agricultural Animal Breeding and Healthy Husbandry, College of Animal Science and Veterinary Medicine, Tianjin Agricultural University, Jinjing Road No. 22, Xiqing District, Tianjin 300384, China; peixingyao@tjau.edu.cn (X.P.); yuyonger1999@163.com (Y.Y.); 2303020127@stu.tjau.edu.cn (C.G.); 2303020148@stu.tjau.edu.cn (J.W.); 2403020133@stu.tjau.edu.cn (R.Z.); 2403020168@stu.tjau.edu.cn (S.L.); 3 Technology Innovation Center for Food Safety Surveillance and Detection (Hainan), Sanya Institute of China Agricultural University, Sanya 572025, China

**Keywords:** zinc oxide nanoparticles, neurotoxicity, risk assessment, protective agent

## Abstract

The expanding application of zinc oxide nanoparticles (ZnO NPs) in consumer products, medicine, and the food industry has raised significant concerns regarding their potential neurotoxicity. This review synthesizes current understanding of the pathways by which ZnO NPs gain access to the central nervous system (CNS), their resulting neurotoxic effects, and the underlying molecular mechanisms. These nanoparticles primarily breach the CNS via translocation across the blood–brain barrier, axonal transport along sensory nerves, and disruption of the microbiota–gut–brain axis. Upon entry, ZnO NPs induce behavioral deficits, including impaired learning, memory, and motor function, alongside pathological brain damage. The neurotoxicity is driven by a multi-faceted mechanism involving mitochondrial dysfunction, oxidative stress, energy depletion, and neuroinflammation, often triggered by the release of Zn^2+^ ions. Furthermore, ZnO NPs can activate diverse cell death pathways, including apoptosis, ferroptosis, and pyroptosis. Critically, their neurotoxic potential is intrinsically linked to their physicochemical properties, such as size and shape. Emerging evidence also suggests that ZnO NP exposure may promote the aggregation of pathological proteins like Tau, thereby potentially increasing the risk for neurodegenerative diseases. Finally, we discuss potential mitigation strategies, such as surface modification and intervention with natural compounds. This review underscores the need for a refined risk assessment of ZnO NPs to ensure their safe deployment.

## 1. Introduction

In recent years, nanotechnology has transformed from applications primarily in industrial machinery to diverse domains such as medical technology, food production, and particularly, animal husbandry [1,2,3]. Propelled by substantial investment in nanomaterials—particularly metal and metal oxide nanoparticles—nanotechnology has flourished, yielding profound socioeconomic benefits [4,5,6]. Among these, zinc oxide nanoparticles (ZnO NPs) have garnered significant attention for their potential in cosmetics, pharmaceuticals, and food products [7,8,9,10]. ZnO NPs are widely used in sunscreens due to their efficiency in scattering and absorbing ultraviolet (UV) radiation [11,12]. Certain green-synthesized ZnO NPs have been applied in cancer therapeutics [13,14,15]. As zinc is an essential trace element [16], ZnO NPs are also commonly utilized as zinc supplements in animal feed additives [17]. Furthermore, driven by their exceptional antibacterial efficacy, ZnO NPs are being actively incorporated into medical materials and food products [18,19]. However, the potential health risks arising from human and animal exposure to nanoparticles through various routes are a growing concern [20,21,22]. Beyond their pervasive dissemination, the reduction in bulk compounds to nanoscale materials fundamentally alters their physicochemical properties and biological interactions. For instance, the small size and high specific surface area of ZnO NPs confer greater reactivity compared to bulk ZnO [23]. Consequently, a critical question is whether ZnO NPs, by virtue of their distinctive nanoscale properties, pose a greater threat to ecosystems and public health than their bulk counterparts, warranting systematic investigation. Recent advances in research have expanded our understanding of nanomaterial toxicity to encompass the central nervous system (CNS)—the primary network integrating sensory input and coordinating motor responses throughout the body [24]. ZnO NPs can interact with the nervous system, elicit neurotoxicity, and thereby mediate brain tissue damage [25,26,27]. Although existing safety assessments have demonstrated the threat ZnO NPs pose to the brain, their mechanisms of neurotoxicity remain incompletely characterized. Therefore, this review analyzes the neuroinvasive properties and neurotoxic mechanisms of ZnO NPs, along with current protective strategies to mitigate their toxicity, aiming to provide a comprehensive understanding of the risks these particles pose to the CNS. This synthesis aims to inform future risk assessments, regulatory decisions, and the development of strategies to mitigate ZnO NP-induced neurotoxicity.

## 2. Principal Pathways for ZnO NP-Mediated Zn Accumulation in the CNS

Biodistribution studies confirm that ZnO NPs penetrate the CNS [28], exhibiting dose-dependent accumulation within specific brain regions in experimental models. In one study, male C57BL/6J mice received a single intratracheal instillation of ZnO NPs at low (3 μg), medium (6 μg), or high (12 μg) doses. Inductively coupled plasma mass spectrometry (ICP-MS) analysis of cerebral cortex tissue revealed a dose-dependent elevation in zinc levels. By day 3 post-exposure, cortical Zn concentrations increased progressively with dosage, from below 600 μg L^−1^ to approximately 700 μg L^−1^ [25]. Moreover, emerging evidence indicates that the nervous system exhibits heightened sensitivity to Zn^2+^. Following dissolution, ZnO NPs may induce neurotoxicity through liberated Zn^2+^ or other bioactive species within the brain [29]. The Zn^2+^ ions released from ZnO NPs can disseminate systemically to various organs, including the brain, while the remaining nanoparticles provide a continuous source of Zn^2+^, leading to progressive bioaccumulation in neural tissue. Current evidence indicates three principal pathways for ZnO NP translocation to the CNS: (1) blood–brain barrier (BBB) penetration [30], (2) sensory neuronal translocation [28,31], and (3) microbiota–gut–brain axis signaling [32].

### 2.1. Blood–Brain Barrier Pathway

**Figure 1:**
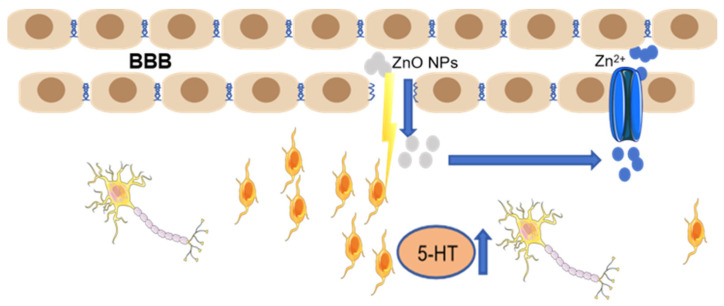
Blood–brain barrier pathway. ZnO NPs compromise the BBB and gain access to the CNS. The subsequent increase in levels of both ZnO NPs and zinc in the brain triggers a rise in 5-HT concentration.

Metal oxide nanomaterials can increase metal levels in the brain parenchyma via four principal mechanisms at the BBB: passive diffusion, inhibition of efflux transporters, receptor-mediated endocytosis, and adsorptive transcytosis [33]. ZnO NPs can cross the BBB and enter the CNS [34]. Repeated intraperitoneal (i.p.) administration of ZnO NPs over 5 days induced significant zinc accumulation in the brains of Swiss mice [35]. This finding is consistent with our earlier observation in SD rats, where a 7-day treatment with ZnO NPs also resulted in marked cerebral zinc enrichment [36]. Furthermore, NP-triggered neuroinflammation can disrupt BBB integrity and increase paracellular permeability, thereby promoting zinc accumulation in the brain parenchyma [37]. Importantly, ZnO NPs that initially cross the BBB via transcytosis can subsequently trigger microglial activation and the release of pro-inflammatory cytokines [38]. This inflammatory response degrades tight junction proteins (e.g., claudin-5), which increases paracellular permeability and creates a vicious cycle that facilitates further zinc influx into the brain parenchyma [39]. Apolipoproteins B and E (ApoB, ApoE) in the circulation can adsorb onto ZnO NPs, facilitating their uptake into brain endothelial cells via receptor-mediated endocytosis. This process mediates the transcytosis of nanoparticles across the BBB [25]. Following intragastric administration in mice, ZnO NPs can translocate systemically from the gastrointestinal tract into the circulation. Subsequent compromise of the BBB facilitates their accumulation in the brain, ultimately elevating levels of 5-hydroxytryptamine (5-HT) [40]. A schematic representation of these key BBB-translocation pathways is provided in Figure 1.

### 2.2. Sensory Neural Translocation Pathway

**Figure 2:**
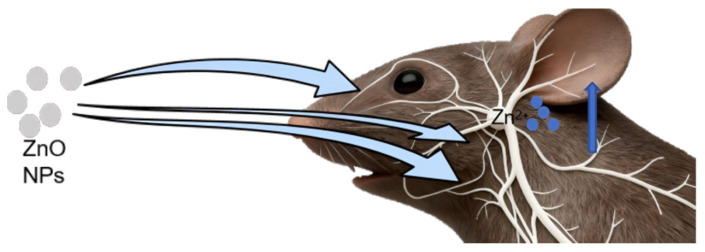
Sensory neural translocation pathway. Intranasal and oral exposure to ZnO NPs enables translocation along neural pathways—including the olfactory, trigeminal, and gustatory nerves—which facilitates zinc accumulation in the brain.

Early epidemiological studies linked occupational inhalation of micrometer-scale ZnO particles during welding to human cognitive impairment, a phenomenon initially attributed to olfactory nerve translocation [41]. This mechanism has now received experimental validation, as contemporary studies confirm that ZnO nanoparticles exploit the nasal–olfactory pathway to reach neural tissue. Both inhaled airborne ZnO nanoparticles and ingested ZnO NP-contaminated materials can stimulate sensory nerves—including the olfactory, trigeminal, and gustatory pathways—enabling axonal transport to the brain. Experimental evidence indicates that inhaled ZnO NPs bypass systemic circulation via two principal routes: (1) trans-epithelial transport across the olfactory mucosa with subsequent uptake by the trigeminal nerve, and (2) direct retrograde axonal transport along olfactory neurons into the brain parenchyma [31,42]. These principal neuro-invasive routes are schematically summarized in Figure 2. In a controlled study, 4-week-old male Sprague Dawley (SD) rats received a single intranasal instillation of ZnO NPs (13 mg Zn/kg), with ZnSO_4_ (at an equimolar Zn concentration) serving as the ionic control. After 7 days, the ZnO NPs had dissolved and transformed, and the resulting Zn^2+^ ions were transported via the olfactory bulb-to-brain pathway, elevating zinc levels in cerebral tissues. X-ray absorption near-edge structure (XANES) spectroscopy revealed comparable Zn speciation and analogous molecular perturbations in the hippocampus of ZnO NP-exposed and ZnSO_4_-control rats. This convergence indicates that the observed neurobiological effects stem predominantly from Zn^2+^ ions released during nanoparticle dissolution, rather than from the particulate form itself [29]. Following alternate-day sublingual administration of ZnO NPs (50–100 nm) for 30 consecutive days in Wistar rats, ICP-MS analysis revealed a significant increase in zinc content in the tongue, gustatory nerves, and brain tissues, with no significant changes observed in the blood or other organs. TEM further showed that ZnO NPs accumulated in close proximity to synaptic regions [28]. In taste exposure experiments, 30 days of sublingual ZnO NP administration (50 mg kg^−1^ d^−1^) resulted in nanoparticle absorption through taste buds and subsequent transport to the brain via gustatory nerves—specifically, the chorda tympani and glossopharyngeal nerves. Transmission electron microscopy (TEM) of the hippocampus revealed vacuolar degeneration of myelinated nerve fibers in both ZnO NP- and ZnSO_4_-exposed groups [43]. Importantly, rats receiving sublingual ZnO NP administration exhibited significantly higher cerebral zinc levels than their intragastrically dosed counterparts, despite equivalent cumulative exposure. This highlights the superior efficiency of gustatory-nerve-mediated transport over gastrointestinal absorption for delivering zinc to the CNS.

### 2.3. Microbiota–Gut–Brain Axis Pathway

**Figure 3:**
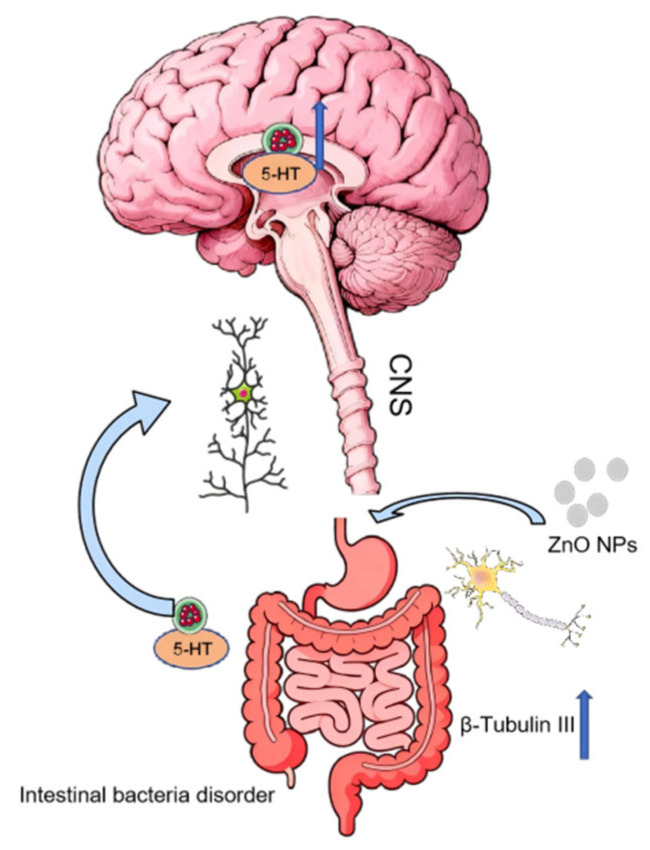
Microbiota–gut–brain axis pathway. ZnO NP exposure induces gut microbiota dysbiosis and activates βIII-tubulin, stimulating enteric neuronal excitation. These excitatory signals (e.g., serotonin, 5-HT) are subsequently propagated to the central nervous system, leading to elevated serotonin levels in the brain.

The gastrointestinal tract is the primary site of interaction for ingested ZnO NPs. Critically, emerging evidence identifies the gut as a key regulator in neurological disorders, wherein nanoparticle-induced dysbiosis and barrier dysfunction can propagate neuroinflammation via the microbiota–gut–brain axis [44,45]. The gut microbiota exhibits high susceptibility to diet-derived xenobiotics—including food additives, pharmaceuticals, and toxicants—which modulate neural function through the microbiota–gut–brain axis. Critically, intestinal dysbiosis triggers hyperexcitation of enteric neurons. These aberrant signals then propagate to the CNS via bidirectional neural pathways, establishing a state of maladaptive communication that can drive neuroinflammatory cascades [46]. First, neural connectivity pathways propagate enteric pathology to the central nervous system. Second, the enteric nervous system (ENS) shares key neurotransmitters with the CNS, such as 5-HT. Consequently, gastrointestinal impairment can disrupt higher-order neural functions, including learning, memory, and motor coordination [47]. Oral exposure to ZnO NPs upregulates the expression of enteric neuron-specific markers, including Hu proteins and β-tubulin III (TuJ1), indicating neuronal activation in the gut. Principal coordinate analysis (PCoA) further indicates that ZnO NPs alter the gut microbial community structure, notably reducing the abundance of Actinobacteria and Bifidobacterium, thereby inducing dysregulation of the microbiota–gut–brain axis. These perturbations drive metabolic reprogramming in the hippocampus and elicit neurobehavioral deficits in young mice [32]. In 4-week-old mice, daily intragastric administration of ZnO NPs (26 mg kg^−1^) for 30 days induced pathological alterations in the gastrointestinal tract. This treatment triggered aberrant enteric neuronal excitation, enhanced intestinal 5-HT biosynthesis and transport, and increased cerebral 5-HT levels via augmented gut–brain communication [40]. Yang et al. revealed that oral exposure to ZnO NPs may lead to neurobehavioral impairments by interfering with the synthesis and secretion of gut-derived melatonin [48]. Collectively, these findings demonstrate that ZnO NP-induced gut dysbiosis compromises neurological function via disruption of the microbiota–gut–brain axis, as specifically evidenced in Figure 3.

## 3. Influence of ZnO NPs’ Nano-Specific Properties on Their Neurotoxicity

### 3.1. Unique Properties of Nanoparticles

Nanoparticles exhibit distinct physicochemical properties compared to their bulk counterparts, including size, morphology, specific surface area, and surface chemistry [49]. Their high specific surface area confers greater reactivity upon ZnO NPs compared to microscale ZnO particles [45,50]. This enhanced reactivity facilitates interactions with biological systems: ZnO NPs can form a protein corona or bind directly to biomolecules via electrostatic, hydrophobic, or hydrogen-bond interactions [45]. Consequently, equimolar exposures to metal oxide nanoparticles, their bulk counterparts, and soluble salts result in distinct toxicological outcomes. Metal oxide nanoparticles, exemplified by ZnO NPs, exhibit greater bioavailability and bioactivity than bulk oxides. Crucially, their toxicity profile proves more pronounced than that of ionic solutions at equivalent molar concentrations [51,52,53]. While non-toxic doses of bulk ZnO are inert, ZnO NPs induce apoptosis in murine microglial cells via activation of the extracellular signal-regulated kinase (ERK) and protein kinase B (Akt) signaling pathways [52]. How et al. found that ZnO NPs can be transferred through the food chain (*E. coli* → *C. elegans*) and that the nanoparticles themselves, rather than the released zinc ions, damage the type D GABAergic motor neurons in C. elegans, resulting in locomotor behavioral deficits [54]. Embryonic exposure of zebrafish (Danio rerio) to ZnO NPs (1–100 mg L^−1^) induces more severe abnormalities than exposure to dissolved Zn^2+^ at equivalent concentrations. ZnO NPs cause more severe disruptions in secondary motor neuron axon outgrowth and dorsal root ganglion development, with neurovascular defects persisting transgenerationally in zebrafish [51]. Upon exposure to ZnO NPs, astrocytes exhibit significantly greater generation of reactive oxygen species (ROS) and increased caspase activity compared to bulk ZnO. Comparative analyses of ZnO NPs, ZnCl_2_, and bulk ZnO reveal that both ZnO NPs and bulk ZnO induce marked mitochondrial damage, whereas ZnCl_2_ had minimal effect on the mitochondrial membrane potential. In colony formation assays, high concentrations (40 and 80 mg L^−1^) of ZnCl_2_ and ZnO NPs significantly reduced astrocyte colony formation, whereas bulk ZnO had little effect. These findings indicate that the potent cytotoxicity of ZnO NPs is not solely attributable to dissolution and ionic release, highlighting a nanoparticle-specific mechanism of neurotoxicity [53].

### 3.2. Relationship Between ZnO NP Properties and Their Neurotoxicity

The toxicological effects of ZnO NPs are closely associated with their physicochemical properties, including size, shape, surface area, and dispersibility [55,56]. Consequently, the toxicity of ZnO NPs must be interpreted in the context of their thorough physicochemical characterization, both in experimental and regulatory settings. For example, ZnO NPs with diameters of 50 and 100 nm exhibit greater toxicity to dopaminergic neurons than their 1000 nm counterparts. This enhanced neurotoxicity may stem from the greater propensity of smaller particles to cross the BBB, their higher cellular uptake, and their larger specific surface area with a higher density of reactive atoms [57]. Zebrafish and SH-SY5Y cells respond differently to various ZnO nanostructures, including nanoparticles (NPs), short nanorods (NRs), and long NRs. At lower concentrations, smaller ZnO NPs and short ZnO NRs demonstrate slightly higher toxicity compared to larger, long ZnO NRs. Conversely, at relatively higher concentrations, long ZnO NRs pose an increased risk. For instance, long NRs can induce Parkinson’s disease (PD)-like symptoms—including motor deficits, dopaminergic neuron loss, and α-synuclein accumulation—to an extent comparable to NPs, primarily via ROS generation. This shift in toxicity profile at higher concentrations may be attributed to the aggregation tendency of smaller ZnO NPs, thereby diluting their size-dependent effects [58]. The relationship between the properties of ZnO NPs and their toxicity varies across biological models [59]. For example, ZnO NRs are more cytotoxic to human lung epithelial cells and cause more pronounced damage in HepG2 cells than ZnO NPs [60,61]. In contrast, ZnO NPs are more toxic to Ana-1 cells than NRs [62]. In three eutrophic freshwater species, ZnO NPs had a lower median lethal concentration (LC_50_) than NRs [59]. Similarly, in marine bivalves, ZnO NPs induced stronger pro-apoptotic and pro-inflammatory effects than NRs [63]. Beyond direct neurotoxicity, ZnO NPs can also be transferred across the placenta, leading to embryonic developmental abnormalities. Studies have shown that exposed mice exhibit neural tube closure defects, which are closely associated with the activation of the endoplasmic reticulum stress–calcium dysregulation–apoptosis pathway [64]. These findings collectively indicate that the comparative risk assessment of ZnO NPs with differing physicochemical properties is model-dependent. Therefore, defining a true safe exposure limit for ZnO NPs requires the integration of detailed physicochemical characterization into the risk assessment framework.

## 4. Neurotoxic Effects of ZnO Nanoparticles

### 4.1. Neurobehavioral Disorders

Neurobehavioral impairments encompass deficits in sensory, motor, and cognitive functions. Rodent behavioral studies demonstrate that ZnO NP exposure induces adverse neurobehavioral outcomes [47]. Amara et al. provided evidence that systemically circulating ZnO NPs can cross the BBB and accumulate in discrete neuroanatomical regions, including the cerebral cortex and cerebellum [65]. Their study using an intravenous (i.v.) rat model provided direct evidence: a concurrent increase in plasma zinc levels and cerebellar Zn^2+^ accumulation, confirming the biodistribution of nanoparticles into the CNS [65]. In 4-week-old Wistar rats, 8 weeks of i.p. ZnO NP exposure impaired spatial cognition, which was mediated by deficits in synaptic plasticity [66]. Similarly, five consecutive days of i.p. ZnO NP administration (300 mg/kg) induced cerebral Zn^2+^ accumulation and anxiety-like behaviors in Swiss mice [35]. Following oral exposure to ZnO NPs (26 mg/kg/day for 30 days), mice exhibited significant behavioral abnormalities, including reduced total distance traveled in the open field test, decreased time spent in the open arms in the elevated plus maze, and impaired balance and coordination in the rotarod test [48]. ZnO NP exposure alters the expression of key neurobehavioral markers such as brain-derived neurotrophic factor (BDNF) and discs large homolog 4 (DLG4). Supporting this, juvenile mice (postnatal day 21) administered ZnO NPs (26 mg kg^−1^) via intragastric gavage for 30 days exhibited deficits in spatial learning and memory in the Morris water maze, as well as suppressed locomotor activity in the open field test [47]. These neurobehavioral impairments may be associated with the upregulation of hippocampus-specific neurobehavioral genes BDNF and DLG4 [47]. Oral exposure to ZnO NPs (50 and 100 mg/kg) induced anxiety-like behaviors in rats, as indicated by reduced time spent in the open arms of the elevated plus maze, and also impaired spatial learning and memory, demonstrated by significantly prolonged escape latency in the Morris water maze test [67]. Following 30-day oral exposure to 34 mg/kg ZnO NPs, mice exhibited reduced total distance traveled and increased anxiety index in the open field test; prolonged immobility time and decreased escape attempts in the tail suspension test; and extended escape latency along with impaired spatial learning and memory in the Morris water maze test [68]. After only 3 days of exposure to an environmentally relevant dose of ZnO NPs (14.6 mg kg^−1^), mice exhibited cognitive impairment in the novel object recognition test. This cognitive deficit was correlated with increased nitric oxide (NO) and thiobarbituric acid reactive substances (TBARS), alongside decreased acetylcholinesterase (AChE) activity [69].

### 4.2. Brain Tissue Injury

Studies indicate that ZnO NP exposure can induce brain tissue damage before observable neurobehavioral deficits manifest [70,71]. Specifically, ZnO NPs have been shown to reduce the brain-to-body weight ratio and induce pathological alterations in the brain tissue of rats. In rats treated with intragastric ZnO NPs, the hippocampus exhibited disorganized architecture and reduced neuronal density. Furthermore, a reduction in Nissl body staining was observed in both the cortex and hippocampus, accompanied by neuronal nuclear shrinkage, collectively indicating significant neuronal injury. Immunohistochemical analysis revealed a marked increase in GFAP immunoreactivity in the cortex and hippocampus. This elevated GFAP expression signifies the transition of glial cells from a quiescent to an activated state [70]. In male albino rats, intragastric (i.g.) administration of ZnO NPs (5.6 mg/kg) induced BBB disruption and cerebellar neurodegeneration, the latter characterized by disorganized neuronal arrangement. Purkinje and granule cells exhibited pathological changes, including Nissl body loss and gliosis. This cerebellar damage was associated with dysregulation of apoptotic and inflammatory proteins: caspase-3 levels were significantly upregulated in Purkinje and granule cells; P53 and COX-2 expression were markedly elevated in cerebellar tissue; and secretion of IL-1β, IL-6, and TNF-α was significantly increased [71]. In Wistar rats orally exposed to ZnO NPs (100 mg/kg) for 75 days, significant neuropathological alterations were observed in the cerebral cortex, including disorganized cortical layers, widespread neuronal loss, multiple fissures and small cysts in the neuropil, shrinkage of pyramidal neurons with perineuronal edema, neuronal degeneration with pyknotic nuclei, as well as vascular dilation and tissue vacuolization. These structural damages were accompanied by elevated levels of TNF-α, IL-6, and p53 in brain tissues [72]. Maternal exposure to ZnO NPs induced neurodegenerative-like alterations in offspring brains, including pyknotic nuclei, eosinophilic cytoplasm, and neuropil vacuolization in hippocampal CA1 neurons, with severity dependent on exposure duration [73].

**Figure 4:**
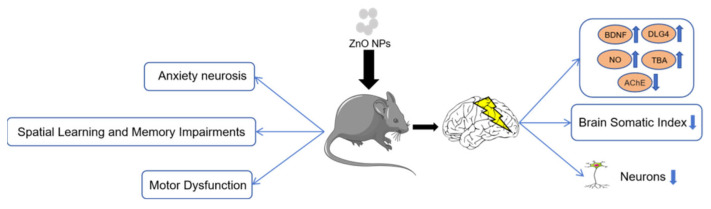
Neurotoxic effects of ZnO nanoparticles. Exposure to ZnO NPs triggered anxiety-like behaviors, spatial learning and memory deficits, and motor impairments in mice. These neurological dysfunctions were associated with marked brain tissue damage, characterized by elevated levels of BDNF, DLG4, NO, and TBA, inhibited AChE activity, a decreased brain somatic index, and a reduction in neuronal cell count.

Based on the aforementioned findings regarding ZnO NPs-induced neurobehavioral disorders and brain tissue injury, we integrated and constructed a mechanistic diagram (Figure 4) to systematically elucidate the underlying molecular and cellular events through which ZnO NPs exposure leads to neurobehavioral impairments and brain tissue damage.

## 5. Mechanisms of ZnO NP-Induced Neurotoxicity

### 5.1. Induction of Neuronal Signaling Dysfunction

ZnO NPs impair neuronal signaling through neurotransmitter dysregulation and disruption of action potential dynamics. Multi-omics analyses revealed that intranasal instillation of ZnO NPs increases acetylcholine (ACh) synthesis, upregulates ACh transporters, and inhibits acetylcholinesterase (AChE) activity in the rat hippocampus, thereby enhancing synaptic ACh levels. The inhibition of AChE may result from Zn^2+^ interactions with the enzyme’s structure or from the transcriptional downregulation of AChE expression [29]. In a study conducted in vitro, ZnO NPs dose-dependently inhibited AChE activity in PC-12 cells [74]. Maternal exposure to ZnO NPs significantly reduced AChE activity in offspring, disrupted acetylcholine metabolism, potentially impaired cholinergic neurotransmission, and consequently may lead to deficits in learning, memory, and other cognitive functions [73]. In the medulla, glutamate is the primary excitatory neurotransmitter underlying respiratory drive. ZnO NPs disrupt glutamatergic neurotransmission by altering glutamate metabolism and inhibiting postsynaptic receptor function. Furthermore, ZnO NPs augment 5-HT release in the mouse cerebral cortex, contributing to memory impairment and locomotor deficits [47]. By disrupting neurotransmission, ZnO NPs impair the normal respiratory drive. In acute experiments using in vitro brainstem-spinal cord preparations from neonatal rats, ZnO NPs compromised the function of respiratory rhythm-generating neurons. This was evidenced by reduced action potential amplitude and peak firing rates, which suppressed synchronized network activity and terminated respiratory rhythmogenesis. This effect may be mediated by Zn^2+^-dependent modulation of neuronal excitability [75].

### 5.2. Induction of Mitochondrial Oxidative Stress and Energy Exhaustion

Mitochondria, the primary energy producers in eukaryotic cells, are essential for mammalian brain function [76,77]. ZnO NPs can induce mitochondrial damage directly or by disrupting the electron transport chain, leading to oxidative stress. This cascade ultimately depletes intracellular ATP levels, thereby altering cerebral energy metabolism [78,79,80]. In rats, intranasal instillation of ZnO NPs upregulated glycolysis but downregulated the tricarboxylic acid (TCA) cycle and oxidative phosphorylation. This metabolic rewiring reduced cerebral ATP levels, creating an energy deficit that culminated in bioenergetic failure [81]. In microglial cells, a 10 h exposure to ZnO NPs (6.6 mg L^−1^) significantly increased intracellular ROS, depolarized the mitochondrial membrane potential, and reduced ATP levels by 50% compared to controls [82]. In astrocytes, treatment with ZnO NPs (5, 20, and 40 mg L^−1^) for 6 h increased ROS levels by 29%, 38%, and 49%, respectively. Alternatively, impaired mitochondrial function may trigger mitophagy, a selective form of autophagy, as a cytoprotective response to mitigate damage. Complementary in vitro studies showed that ZnO NPs are internalized by rat C6 glial cells and induce oxidative stress in a time-dependent manner, evident at 3 and 6 h post-exposure [83]. In SH-SY5Y cells, exposure to ZnO NPs induced mitochondrial swelling and cristae disappearance, significantly reduced mitochondrial membrane potential, and disrupted both mitochondrial structure and function. This led to excessive production of ROS, decreased superoxide dismutase (SOD) activity, elevated malondialdehyde (MDA) concentration, and reduced glutathione (GSH) levels, collectively triggering increased intracellular oxidative stress [64]. Exposure to ZnO NPs disrupts GSH metabolism in nematodes, leading to a reduction in GSH content and affecting the activities of glutathione peroxidase (GSH-Px) and glutathione S-transferase (GSH-ST) [84]. In zebrafish larvae and C. elegans, ZnO NP exposure triggered the accumulation of ROS and superoxide anion (O_2_•^−^). This oxidative burden led to elevated MDA levels, indicating lipid peroxidation, while concurrently depleting GSH and suppressing the activities of key antioxidant enzymes (SOD, catalase (CAT), and GSH-Px) [85]. Interestingly, residual PINK1 recruited parkin from the cytosol to mitochondria, triggering a compensatory mitophagic response that alleviated ZnO NP-induced cytotoxicity [86]. ZnO NPs may contribute to neurodevelopmental and neurodegenerative pathologies by promoting neuroinflammation, microglial activation, ROS accumulation, and neuronal loss [87].

### 5.3. Induction of Neuroinflammation

In contrast to direct neuronal damage, neuroinflammation plays a predominant role in the pathogenesis of neurodegenerative diseases such as Alzheimer’s disease, PD, and amyotrophic lateral sclerosis [88]. This activation inhibits the production and migration of cranial neural crest cells (CNCCs), thereby impairing cranial neural crest development and leading to craniofacial defects in chicken embryos [89]. Following i.g. administration of ZnO NPs (600 mg kg^−1^), rats exhibited elevated serum levels of TNF-α, IL-1β, IL-6, C-reactive protein (CRP), CAT, and GSH. Concurrently, the cerebral cortex showed significant upregulation of the pro-inflammatory mediators TNF-α, IL-1β, and nitric oxide synthase 2 (NOS2) [90]. Sublingual ZnO NP exposure elevated cortical levels of TNF-α, IL-1β, IL-4, IL-6, and IL-13 in a dose-dependent manner, leading to localized neuroinflammation in mice [28]. qRT-PCR analysis showed that ZnO NPs stimulation potently increased the secretion of TNF-α and IL-1β from BV-2 microglial cells, with levels peaking at 3 and 6 h post-exposure. This inflammatory response was orchestrated by the coordinated activation of the NF-κB signaling pathway and the phosphorylation of ERK and p38 MAPKs [70]. In PC-12 cells, ZnO NPs (≥10 μg/mL) elevated the levels of pro-inflammatory cytokines IL-6 and TNF-α in time- and concentration-dependent manners [74]. In SH-SY5Y neuroblastoma cells, ZnO NPs activate the NF-κB signaling pathway and suppress the expression of ubiquitin C-terminal hydrolase L1 (UCH-L1). Conversely, UCH-L1 overexpression deubiquitinates the endogenous NF-κB inhibitor IκB-α, thereby blocking NF-κB nuclear translocation and mitigating neuronal damage [57]. Oral administration of ZnO NPs (400 mg/kg) significantly elevated serum levels of pro-inflammatory cytokines (IL-1β, IL-6, IL-8, and TNF-α) in rats and concurrently induced cerebellar astrocyte proliferation (indicated by increased GFAP expression), thereby establishing a vicious cycle between inflammation and oxidative stress [91].

### 5.4. Induction of Neuronal Apoptosis

Seven-day consecutive exposure to ZnO NPs (40 and 100 mg kg^−1^) induced a dose-dependent upregulation of the apoptotic executioner caspase-3 in rat brain tissue, accompanied by significant DNA fragmentation—a hallmark of programmed cell death [92]. Treatment of SH-SY5Y neuroblastoma cells with 15 μmol L^−1^ ZnO NPs for 12 and 24 h significantly promoted apoptosis via activation of the Akt/caspase-3/caspase-7 pathway. Acridine orange/ethidium bromide (AO/EB) staining and quantitative apoptosis assays confirmed ZnO NP-induced programmed cell death in rat astrocytes. Caspase-3/7 activity increased in a concentration-dependent manner, with 80 mg L^−1^ ZnO NPs triggering a pronounced increase of 71.9 ± 3.8% compared to controls [93]. ZnO NPs (3–12 μg/mL, 24 h) induced mitochondrial-mediated apoptosis in SH-SY5Y cells, characterized by Caspase-3/9 activation, Bax/Bcl-2 dysregulation, and dose-dependent apoptotic rate elevation [94]. In the rat cerebellum, Bax expression was elevated, and Calbindin D28k level was reduced following ZnO NP exposure (400 mg/kg), resulting in calcium dyshomeostasis and enhanced apoptosis of Purkinje cells [91].

### 5.5. Induction of Neuronal Ferroptosis

Ferroptosis is an iron-dependent form of regulated cell death characterized by inactivation of glutathione peroxidase 4 (GPX4), iron dyshomeostasis, and lethal lipid peroxidation [95,96,97]. Nanoparticles have been reported to suppress tumor growth by inducing ferroptosis in mouse xenograft models, establishing a link between nanomaterial exposure and this cell death pathway [98,99]. Emerging evidence demonstrates that ZnO NPs can induce ferroptosis. Following intratracheal instillation of ZnO NPs, mouse cortical neurons exhibited features of ferroptosis, including significantly decreased expression of the key regulators GPX4 and solute carrier family 7 member 11 (SLC7A11). Conversely, expression of the ferroptosis-promoting protein voltage-dependent anion channel 3 (VDAC3) was markedly increased. In neuron-like PC-12 cells, ZnO NP treatment induced concentration-dependent decreases in GPX4 and SLC7A11 protein levels, confirming ferroptosis in vitro. Mechanistically, the JNK pathway mediated ZnO NP-induced lipid peroxidation and ferroptosis, as the JNK inhibitor SP600125 reversed these effects. The specific ferroptosis inhibitor ferrostatin-1 attenuated ZnO NP-induced neuronal death in vivo and in vitro, confirming ferroptosis as a key mechanism of ZnO NP neurotoxicity [25]. In bEnd.3 cells, treatment with 20 μg/mL ZnO NPs for 9 h induced lysosomal NP accumulation, increased intracellular iron and lipid peroxidation, and downregulated GPX4, culminating in oxidative stress and ferroptosis [38]. This intracellular iron overload dysregulated autophagic flux and increased membrane permeability, indicating compromised blood–brain barrier integrity and exacerbated cytotoxicity [38].

### 5.6. Induction of Neuronal Pyroptosis

Pyroptosis is a pro-inflammatory form of regulated cell death characterized by plasma membrane rupture and release of intracellular contents following activation of intense inflammatory cascades by intrinsic or extrinsic stimuli [100]. Emerging evidence suggests a potential link between ZnO NPs and pyroptotic cell death. Exposure of BV-2 microglial cells to 80 mg L^−1^ ZnO NPs significantly reduced cell viability and induced intracellular ROS accumulation and mitochondrial damage. Flow cytometric analysis using Annexin V-FITC/PI staining detected no significant population of early apoptotic cells (Annexin V^+^/PI^−^). Western blot analysis confirmed the absence of poly (ADP-ribose) polymerase (PARP) cleavage and caspase-3 activation. In contrast, the late apoptotic/necrotic cell population (Annexin V^+^/PI^+^) accounted for 68.2 ± 6.7% of the total. This biomarker profile, together with observed plasma membrane damage, suggests that ZnO NPs induce non-apoptotic, membrane-disruptive cell death, potentially through pyroptosis or secondary necrosis [101].

## 6. Potential Risk of ZnO NP-Induced Neurodegenerative Disorders

Neurodegenerative diseases represent a growing public health challenge with substantial societal burdens. Epidemiological studies demonstrate a positive correlation between nanoparticles and neurodegenerative diseases [102,103,104]. For instance, titanium dioxide nanoparticles (TiO_2_ NPs) are an established risk factor for PD, capable of inducing motor deficits, neuronal loss, dopamine depletion, and Lewy body aggregation [105,106]. Emerging evidence indicates that ZnO NPs pose a neurodegenerative risk by inducing pathological aggregation of the Tau protein. Exposure to ZnO NPs triggers Tau aggregation and generates proteolytic fragments in SH-SY5Y cells, recapitulating key molecular features of neurodegenerative pathology [58]. The study by Chuang et al. demonstrated that the neurobiological effects of ZnO NPs are concentration- and property-dependent [107]. At low concentrations, ZnO NPs exhibit neuroprotective effects by inhibiting protein oxidation, activating autophagic flux, and suppressing Tau protein aggregation. In contrast, high concentrations inactivate autophagic flux and promote Tau aggregation in the brain. In their model, 7-week-old male SD rats exposed to ZnO NPs (10 mg kg^−1^) exhibited microglial activation in the hippocampus and elevated Tau expression in the cerebellum and hippocampus. Under physiological conditions, accumulated Tau protein in the healthy brain is efficiently cleared by degradation mechanisms such as autophagy. However, high-concentration ZnO NP exposure did not significantly alter key hippocampal autophagy markers, such as LC3-II and p62. This absence of autophagic response—contrary to previously reported ZnO NP-mediated autophagy induction [107]—demonstrates a failure to upregulate cerebral autophagy under neurotoxic conditions. Consequently, this compromised degradation capacity promotes further pathological Tau accumulation.

## 7. Approaches for Reducing ZnO NP-Induced Toxicity

Current strategies to mitigate ZnO NP toxicity remain limited, primarily falling into two categories: surface modification to reduce their intrinsic reactivity and the use of protective natural compounds. Coating ZnO NPs with bovine serum albumin (BSA) via covalent or non-covalent interactions reduced their cytotoxicity and suppressed ROS generation [108]. Unlike bare ZnO NPs, incorporation into sodium alginate–gum acacia hydrogels prevented red blood cell aggregation. In Vero cells, this hydrogel formulation significantly reduced MDA production, indicating attenuated oxidative stress and consequently lower cytotoxicity [109]. Oral administration of betaine alleviated ZnO NP-induced depressive-like behaviors in mice. Betaine treatment also reduced serum MDA levels and enhanced the activities of SOD and glutathione peroxidase (GPx). Furthermore, betaine treatment ameliorated histological alterations in the hippocampus [110]. In SD rats, intraperitoneal injection of Apis mellifera venom significantly alleviated ZnO NP-induced depression, anxiety, memory impairment, and spatial learning deficits. The treatment also reduced serotonin and dopamine levels, as well as zinc content, in brain tissue. Additionally, it attenuated the ZnO NP-induced increase in neurofilament and GAP-43 immunoreactivity [111]. In Drosophila, dietary quercetin and astragaloside supplementation during the reproductive phase ameliorated ZnO NP-induced developmental toxicity. This protection was mediated by the attenuation of oxidative stress—via upregulation of GSH and SOD and downregulation of MDA—and the subsequent suppression of ferroptosis [112]. Oral administration of saffron extract (200 mg/kg, 21 days) attenuated ZnO NP-induced neurotoxicity by enhancing antioxidant enzyme activities and GSH levels while reducing lipid peroxidation, suppressing neuroinflammation via decreased hippocampal IL-6 and IL-1α, and preserving neurobehavioral and cholinergic function [67]. Thymoquinone and quercetin significantly reduced ZnO NP-induced chromosomal aberrations, micronucleus formation, and DNA damage. Additionally, they enhanced the activities of SOD and catalase (CAT), decreased LPO levels, and increased GSH content in the liver. The optimal protective doses of thymoquinone and quercetin against ZnO NP-induced toxicity were determined to be 18 mg/kg body weight and 500 mg/kg body weight, respectively [112]. Quercetin (50 mg/kg/d) effectively mitigates ZnO NP-induced cerebellar toxicity. The mechanism involves inhibiting oxidative stress (reducing MDA and TOS, increasing SOD, GSH, and TAC), alleviating inflammatory response (downregulating IL-1β, IL-6, IL-8, TNF-α, and GFAP), maintaining calcium homeostasis, and reducing apoptosis (downregulating Bax and upregulating Calbindin D28k), thereby preserving the structural and functional integrity of Purkinje cells [91]. Folate pretreatment (10 mg/kg, i.p., one week) alleviates ZnO NP-induced neurotoxicity by increasing serum GFAP and MBP levels, decreasing MAOA, and improving astrocyte function and myelin integrity [113]. In our previous study, we demonstrated that paeoniflorin restored ZnO NP-induced gut microbiota dysbiosis by increasing the relative abundance of beneficial bacteria and reducing harmful bacteria. Furthermore, paeoniflorin ameliorated ZnO NP-induced hepatic metabolic disorders, including improvements in organic acid and lipid metabolism. It also alleviated abnormalities in body weight, liver index, hepatic histopathology, and biochemical markers, while suppressing pro-inflammatory cytokines and oxidative stress products. Mechanistically, paeoniflorin regulated the SIRT1–mTOR–TFEB axis, thereby inhibiting ZnO NP-induced hepatocyte pyroptosis [114].

## 8. Conclusions and Future Perspectives

ZnO NPs induce neurotoxicity primarily by elevating Zn^2+^ levels in the brain, which they access via multiple routes, including translocation across the BBB, sensory neural translocation, and the microbiota–gut–brain axis. The neurotoxicity of ZnO NPs exhibits nanoparticle-specific characteristics that are intrinsically linked to their physicochemical properties, such as size, shape, and surface chemistry. Experimentally, ZnO NPs cause significant brain tissue damage and lead to neurobehavioral impairments in animal models. The mechanisms underlying ZnO NP neurotoxicity are multifaceted, involving disrupted neuronal signaling, oxidative stress, neuroinflammation, and the activation of diverse cell death pathways, including apoptosis, ferroptosis, and pyroptosis. These processes collectively contribute to the pathogenesis of neurodegenerative diseases. These effects are mediated by complex signaling networks, including NF-κB, MAPK, BDNF, PINK1, and caspase-3. However, key aspects of their neurotoxicology remain poorly understood, including the precise transformation processes and chemical speciation of ZnO NPs within the nervous system. Research on ZnO NP-induced neuronal death, particularly non-apoptotic forms like pyroptosis and ferroptosis, remains in its infancy. Furthermore, most studies have focused on neurons, with the roles of glial cells receiving considerably less attention. Therefore, future studies are warranted to elucidate the detailed molecular mechanisms and to explore potential links to a broader range of neurological conditions, including cerebral ischemia, traumatic brain injury, Huntington’s disease, and amyotrophic lateral sclerosis. Current strategies to mitigate ZnO NP toxicity—primarily surface modification and the use of natural protective compounds—remain limited. Given the current irreplaceability of ZnO NPs in many applications, expanding the search for effective natural protective agents is imperative. Concurrently, future efforts must prioritize the development of safer-by-design production protocols, rigorous dosage control, and stringent regulatory frameworks to minimize the risks associated with ZnO NP exposure.

## Data Availability

The original contributions presented in this study are included in the article.

## Abbreviations

The following abbreviations are used in this manuscript:

ACh	Acetylcholine
AChE	Acetylcholinesterase
Akt	Protein kinase B
ApoB	Apolipoprotein B
ApoE	Apolipoprotein E
BBB	Blood–brain barrier
BDNF	Brain-derived neurotrophic factor
CAT	Catalase
CNS	Central nervous system
CRP	C-reactive protein
DLG4	Disc large homolog 4
ENS	Enteric nervous system
GFAP	Glial fibrillary acidic protein
GPX4	Glutathione peroxidase 4
GSH	Glutathione
GSH-Px	Glutathione peroxidase
GSH-ST	Glutathione S-transferase
ICP-MS	Inductively coupled plasma mass spectrometry
i.g.	Intragastric
IL-1β	Interleukin-1 Beta
IL-6	Interleukin-6
i.p.	Intraperitoneal
i.v.	Intravenous
MAOA	Monoamine oxidase A
MBP	Myelin basic protein
MDA	Malondialdehyde
NOS2	Nitric oxide synthase 2
PARP	Poly (ADP-ribose) polymerase
PCoA	Principal coordinate analysis
SOD	Superoxide dismutase
TAC	Total antioxidant capacity
TBARS	Thiobarbituric acid reactive substances
TEM	Transmission electron microscopy
TNF-α	Tumor necrosis factor-alpha
UCH-L1	Ubiquitin C-terminal hydrolase L1
UV	Ultraviolet
VDAC3	Voltage-dependent anion channel 3
XANES	X-ray absorption near-edge structure
ZnO NPs	Zinc oxide nanoparticles
α-synuclein	Alpha-synuclein
5-HT	5-Hydroxytryptamine
